# Diphenyl Difluoroketone: A Potent Chemotherapy Candidate for Human Hepatocellular Carcinoma

**DOI:** 10.1371/journal.pone.0023908

**Published:** 2011-08-25

**Authors:** Yingjian Liang, Dalong Yin, Limin Hou, Tongsen Zheng, Jiabei Wang, Xianzhi Meng, Zhaoyang Lu, Xuan Song, Shangha Pan, Hongchi Jiang, Lianxin Liu

**Affiliations:** 1 Key Laboratory of Hepatosplenic Surgery, Department of General Surgery, The First Affiliated Hospital of Harbin Medical University, Ministry of Education, Harbin, Heilongjiang Province, People's Republic of China; 2 Department of Emergency Surgery, The First Affiliated Hospital of Harbin Medical University, Harbin, Heilongjiang Province, People's Republic of China; University of Illinois at Chicago, United States of America

## Abstract

Diphenyl difluoroketone (EF24), a molecule having structural similarity to curcumin, was recently reported to inhibit proliferation of various cancer cells significantly. Here we try to determine the effect and mechanism of EF24 on hepatocellular carcinoma. 2 µM EF24 was found to inhibit the proliferation of PLC/PRF/5, Hep3B, HepG2, SK-HEP-1 and Huh 7 cell lines. However, even 8 µM EF24 treatment did not affect the proliferation of normal liver LO2 cells. Accordingly, 20 mg/kg/d EF24 inhibited the growth of the tumor xenografts conspicuously while causing no apparent change in liver, spleen or body weight. In addition, significant apoptosis and G_2_/M phase cell cycle arrest were found using flow cytometry. Besides, caspases and PARP activation and features typical of apoptosis including fragmented nuclei with condensed chromatin were also observed. Furthermore, the mechanism was targeted at the reduction of nuclear factor kappa b (NF-κB) pathway and the NF-κB–regulated gene products Bcl-2, COX-2, Cyclin B1. Our study has offered a strategy that EF24 being a therapeutic agent for hepatocellular carcinoma.

## Introduction

Hepatocellular carcinoma (HCC) is a common solid organ malignancy worldwide, with about 600,000 new cases diagnosed each year [1], [2], [3], [4]. Surgical resection, in the form of partial hepatectomy or total hepatectomy followed by liver transplantation may provide an occasional incidence of cure. However, it can be performed only in selected patients whose tumors are small and away from major vessels and have not metastasized to extrahepatic organs [5], [6], [7]. In general, patients with unresectable HCC have a dismal prognosis, and actually, these patients do not benefit much from nonsurgical treatments, such as systematic chemotherapy or chemoembolization [8], [9], [10], [11], [12], [13].

**Figure 1:**
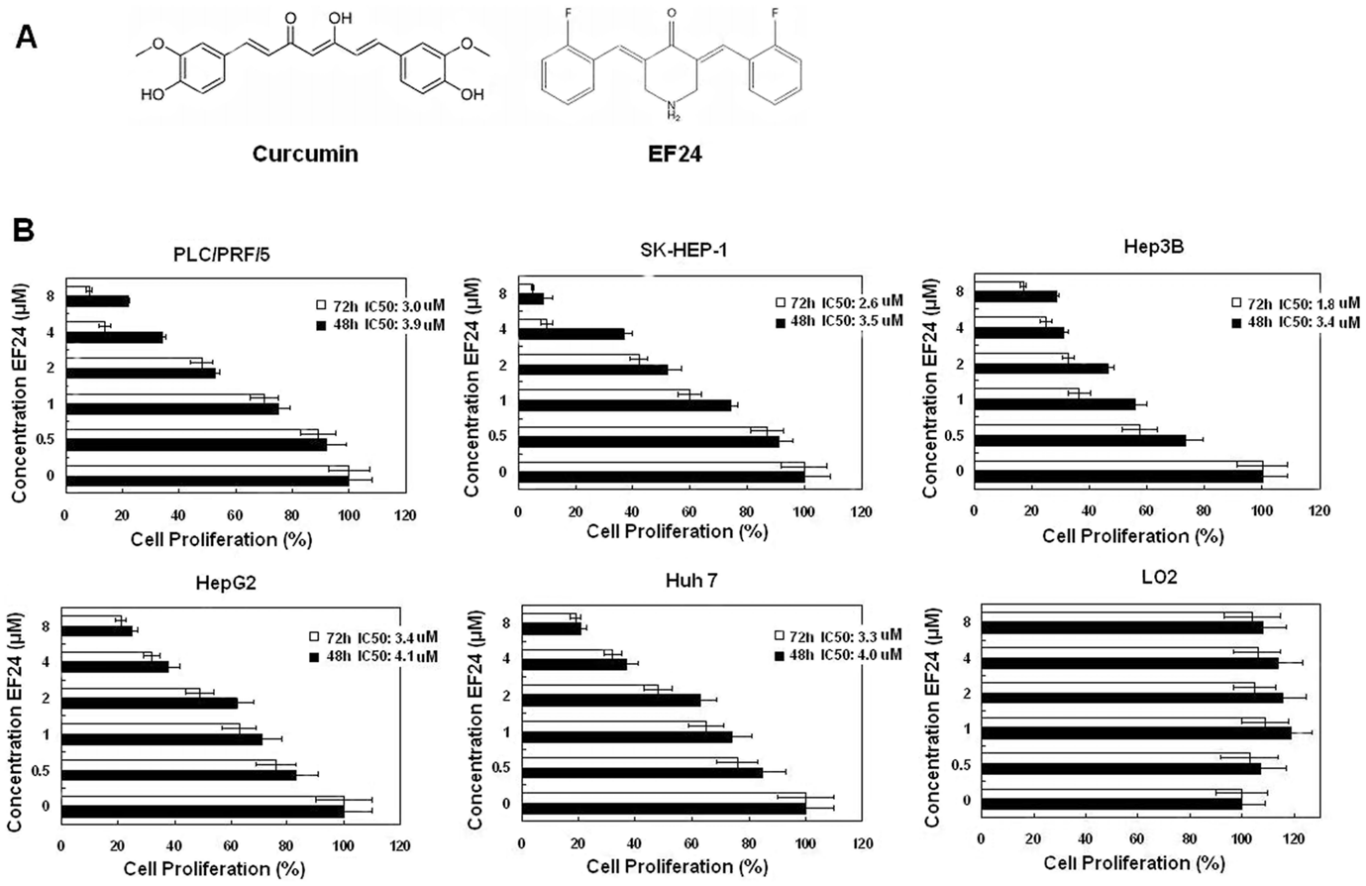
EF24 inhibited liver cancer cell proliferation. (**A**). The topological structures of curcumin (diferuloylmethane) and EF24 (diphenyl difluoroketone). (**B**). EF24 treatment on proliferation of PLC/PRF/5, SK-HEP-1, Hep3B, HepG2, Huh 7 and LO2 cells. These cells were incubated with increasing doses of EF24 (0.5–8 µmol/L) for 48-h and 72-h periods and analyzed for cell proliferation using cell counting kit-8 assay.

Systemic chemotherapy has been tested and shown to be minimally effective in HCC treatment due to toxicity to normal cells and chemoresistance [14]. Based on data from previous studies [15], Doxorubicin is generally considered to be the first-line treatment for HCC, however this drug used alone has shown a response rate only between 20 to 30% [16] and is associated with multiple adverse events and drug resistance. As a result, the search for more effective chemotherapeutic agents is still ongoing, and new regimens are under active investigation. Previously, lots of studies have examined the anticarcinogenic activity of curcumin in HCC. Curcumin has been found to interrupt the cell cycle, have cytotoxic effects, and have a role in antiproliferation and induction of apoptosis in many hepatocellular carcinoma cell lines [17]. One proposed mechanism for curcumin's inhibition of tumor growth in HCC is the induction of apoptosis via a caspase cascade [18]. Another proposed mechanism of curcumin is through the inhibition of hypoxia-inducible factor-1 by degrading the aryl hydrocarbon receptor nuclear translocator [19], [20]. Further, it has been shown that mitochondrial hyperpolarization is a prerequisite for curcumin induced apoptosis and DNA damage is the initial event in a chain leading to apoptosis in HepG2 cells [21]. Moreover, curcumin could prevent the induction of hepatic hyper plastic nodules, body weight loss, and hypoproteinemia in carcinogen induced as well as xenograft hepatic cancer models. A considerable number of reports have also described the anticancer effects of curcumin on HCC in vivo. One of these studies suggested that curcumin had anticarcinogenic effects mediated through the reduction of COX-2 and VEGF [22]. However, one potential problem with the clinical use of curcumin is its low potency and poor absorption characteristics[23]. In an attempt to retain curcumin's favorable medicinal properties and safety profile while increasing its potency, one analog of curcumin (EF24) (Fig. 1A) was synthesized and applied to many cancer cell types. Sun et al. found that the cytotoxic effect of EF24 against MDA-MB-231 human breast cancer, RPMI-7951 human melanoma and DU-145 human prostate cancer cells arises, at least in part, from the induction of cell cycle arrest and subsequent apoptosis by means of a redox-dependent mechanism and EF24-tripeptide chloromethyl ketone drug delivery system could increase the effect of EF24 [24]. Thomas *et al.* found that treatment of MDA-MB231 breast and PC3 prostate cancer cells with EF24 could lead to inhibition of HIF transcriptional activity [25]. The studies of Dharmalingam *et al.* showed that EF24 treatment of HCT-116 and HT-29 colon and AGS gastric adenocarcinoma cells could result in growth inhibition without affecting the proliferation of normal human fibroblasts [26]. Thomas et al. have found that EF24-induced decease of lung cancer cell viability was accompanied by upregulated mitogen-activated protein kinases (MAPK) as evidenced by increased phosphorylation of ERK1/2, JNK, and p38 [27]. These results suggested that the novel curcumin-related compound EF24 is a potent antitumor agent [24], [28], [29]. Furthermore, the fact that the feasibility of using this drug in HCC treatment yet has not been studied drew our attention. Therefore, the objective of the current study is to investigate the in vivo and in vitro anticancer potential of EF24 and delineate the underlying mechanisms.

In this article, the results of our experiments indicated that EF24 could potently inhibit HCC cell proliferation and induce apoptosis *in vitro* and vivo. In addition, G_2_/M arrest of liver cancer cells was also observed. More importantly, we provide evidences that the molecular mechanisms of the action of EF24 are possibly by inhibiting the nuclear factor kappa b (NF-κB) pathway, coupled with the reduction of the expression of NF-κB regulated genes, including Bcl-2, cyclooxygenase-2(COX-2) and CyclinB1. In vivo study has also shown that tumor growth was significantly suppressed after EF24 treatment.

## Results

### EF24 inhibits liver cancer cell proliferation

First, we determined the effects of EF24 on cell proliferation of liver cancer cell lines. We used five HCC cell lines with different p53 status (HepG2 and SK-HEP-1 with wt p53; PLC/PRF/5 and Huh 7 with p53 mutation; Hep3B with null p53) which are widely used in the liver cancer research to investigate the effects of EF24 on HCC. We also use a LO2 cell line to see whether EF24 has the same effects on normal liver cells. Our data presented here show that EF24 significantly suppressed proliferation of all the liver cancer cell lines within a 48 h period, which continues to 72 h (Fig. 1B). More importantly, the effects were observed at a dose of nearly 2 µmol/L, a dose at which curcumin had no significant effect on cancer cell proliferation [26].

### EF24 induces G_2_/M cell cycle arrest in liver cancer cells

**Figure 2:**
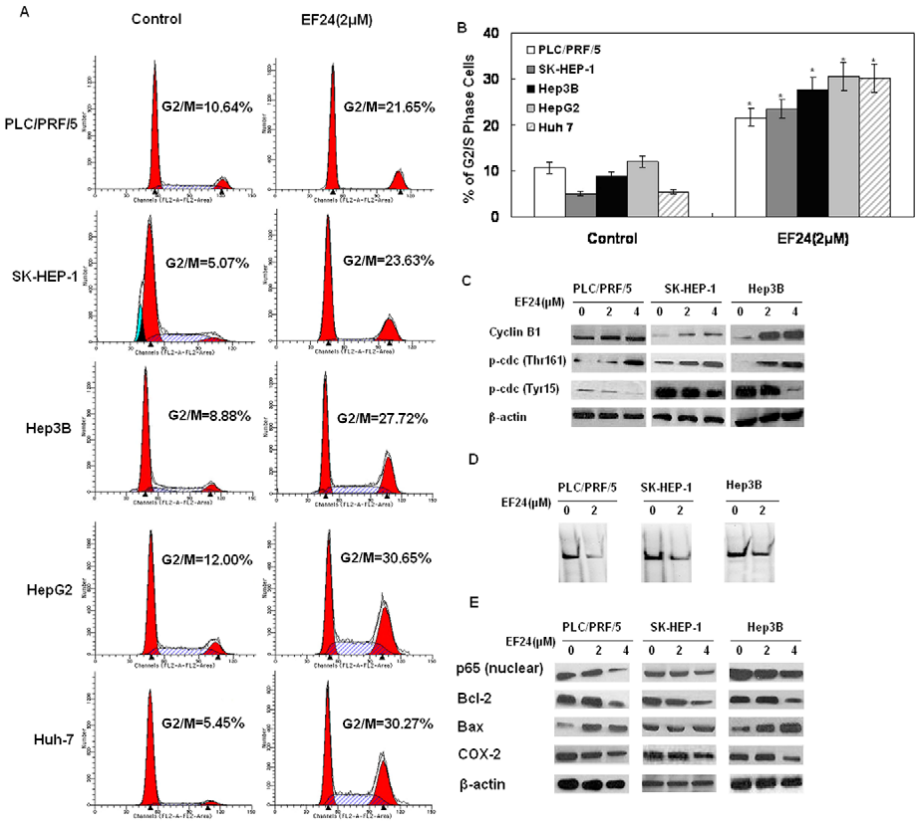
EF24 induced cell cycle arrest and inhibited the NF-κB pathway. (**A**). DNA content (propidium iodide) and cell cycle analysis of EF24-treated cells. The five HCC cells were incubated with 0, 2 µmol/L EF24 for 48 h. The numbers of cells in G0/G1 phase, S phase and G2/M phase was determined via flow cytometry. (**B**). Representative histograms from cytometrically analyzed the five cell lines treated with control and EF24. Assay was done in triplicate and p<0.05 is denoted by “*”. (**C**). Expression of G_2_/M cell cycle relative proteins Tyr15 and Thr161 phosphorylation of cdc2 and Cyclin B1 were determined via western blot after treatment with 2 and 4 µmol/L EF24 for 24 h. β-actin was used as the internal control. (**D**). Nuclear extracts were prepared and subjected to EMSA to measure NF-κB DNA-binding activity. (**E**). Expression of p65, Bcl-2, Bax and COX-2 via western blot.

To determine whether the growth inhibition of liver cancer cells by EF24 was caused by cell cycle arrest or apoptosis, the cells were treated with 2 µmol/L EF24 for 48 h. The cells were then fixed, and cell cycle populations were determined by flow cytometry. The results showed that EF24 induced G_2_/M arrest significantly (Fig. 2A, 2B). The western blot results indicated that the expression of G_2_/M cell cycle regulating factors Cyclin B1 and Tyr15 phosphorylation of cdc2 showed a time-dependent decrease with increasing dose of EF24. On the other side, increases of Thr161 phosphorylation of cdc2 were observed in the same conditions (Fig. 2C). These data suggests that the inhibition of cell proliferation by EF24 is associated with the induction of G_2_/M phase arrest.

### EF24 potently suppresses the NF-κB signaling pathway and down-regulates the expression of NF-κB regulated gene products in vitro and vivo

Previous reports demonstrated that curcumin could inhibit many tumors' growth by targeting the nuclear factor-κB pathway [30], hereby, we investigated whether the effect of EF24 on cancer cells is associated with the inhibition of NF-κB activation. NF-κB DNA-binding activity was examined by EMSA. As shown in Fig. 2D, EF24 markedly reduced NF-κB DNA-binding activity compared to control in all liver cancer cell lines. Furthermore, the western blotting data showed an obvious down-regulation of nuclear p65 activation after exposed to different concentration of EF24 (2 µM, 4 µM) as shown in Fig. 2E. NF-κB is known to regulate the expression of COX-2 (involved in proliferation). Western blotting (Fig. 2E) revealed significant reductions in the expression of COX-2 in the three cell lines from the EF24-treated groups compared with those from the control group. We also assessed the expression of other NF-κB–regulated genes Bcl-2, Bax and Cyclin B1, the overexpression of which have been linked to tumor survival, apoptosis and cell cycle arrest [31], [32], [33], [34]. Western blotting revealed that all the HCC cells exposed to 4 µM EF24 have shown a reduction of Bcl-2 and Cyclin B1, and a concomitant increase of Bax compared with the control cells (Fig. 2E).

### EF24 Induces Apoptosis in liver cancer cells

**Figure 3:**
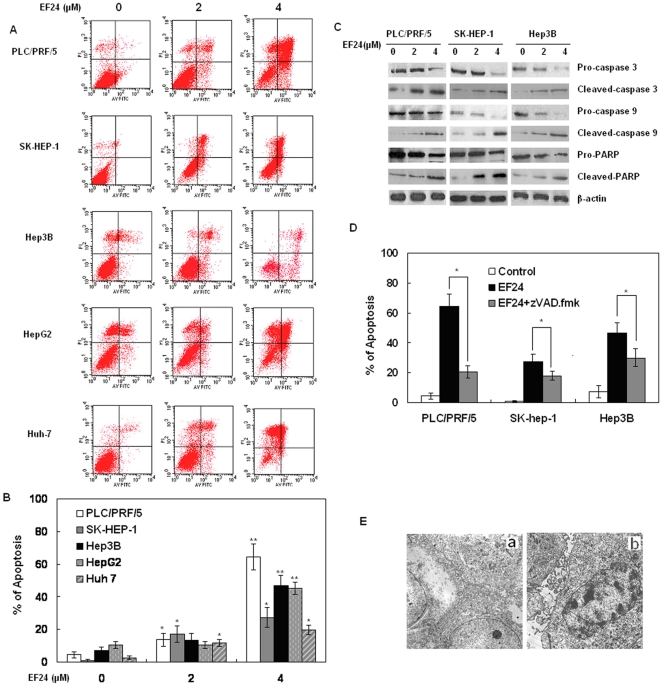
EF24 induced cancer cell apoptosis. (**A**). Five liver cancer cells were treated with 0, 2 and 4 µmol/L EF24 for 48 h and harvested. Flow cytometry was performed to observe apoptosis rates. (**B**). Representative histograms from cytometrically analyzed the five cell lines treated with control (DMSO) and EF24. Assay was done in triplicate. *p<0.05; **p<0.01. (**C**).The target proteins were detected by Western blot analyses. (**D**). Histograms from three cell lines treated with EF24 and EF24 in combination with pan-caspase inhibitor (z-VAD-fmk). (**E**). Electron microscopic findings in PLC/PRF/5 cancer cells treated with EF24. Cancer cells without treatment exhibited innumerable microvilli and well developed filopodia on the cell surface with intact nuclei (**a**). In contrast, EF24 induced distinct changes on the cell surface with decreased filopodias and microvilli, and changes in nuclei (**b**). Original magnification: ×6,000 in a; ×8,000 in b.

To further investigate the underlying mechanism of decreased cell proliferation observed in the CCK-8 assay, we examined the apoptosis effect on liver cancer cells induced by EF24 using Annexin V/propidium iodide assay as described in materials and methods section. As shown in Fig. 3A and 3B, all of the five liver cancer cell lines have shown a concentration-dependent apoptosis, including early as well as late apoptotic cell death. The analysis demonstrated >45% of the Hep3B cells apoptosis within 48 h after initiation of EF24 treatment (4 µM), whereas >60% of the cancer cells in PLC/PRF/5. Then we further determined the levels of apoptosis-related proteins in cells treated with EF24. As shown in Fig. 2E, the PLC/PRF/5, Hep3B and SK-Hep-1 cells exposed to EF24 have shown a concentration-dependent reduction of Bcl-2 and a concomitant increase of Bax compared with the control cells. The contribution of cell death pathway in EF24-treated liver cancer cells was also investigated. The expression levels of caspases were also examined by Western analysis. The analysis demonstrated that caspase-3 was cleaved into fragments after exposure to EF24, and cleavage of caspase-3 became more intense with increased concentrations of EF24. A similar tendency was observed for caspase-9. Activation of caspase-3 was further confirmed by poly (ADP-Ribose) polymerase (PARP) cleavage, a typical feature of caspase-dependent apoptosis. PARP activation was also found as is shown in Figure 3C. These results suggest that EF24 induced the apoptosis of HCC cells at least partly by activating caspases and promoting PARP cleavage. The selective pan-caspase inhibitor z-VAD-fmk was used to determine whether EF24-induced apoptosis of liver cancer cells was caspase-dependent. As is shown in Fig. 3D, EF24–induced apoptosis was partly inhibited by the pan-caspase inhibitor (z-VAD-fmk) in PLC/PRF/5, Hep3B and Sk-Hep-1 cell lines.

### Morphological changes in PLC/PRF/5 cells analyzed by electron microscopy

PLC/PRF/5 cells without EF24 treatment exhibited innumerable microvilli and well-developed filopodia on the cell surface with intact nuclei (Fig. 3Ea). However, cancer cells treated with EF24 at a concentration of 2 µM demonstrated distinct changes on the cell surface with decreased filopodias and microvilli accompanied by secretory vesicle formation. Furthermore, 2 µM EF24 induced features typical of apoptosis including fragmented nuclei with condensed chromatin (Fig. 3Eb).

### EF24 inhibits in vivo tumor growth and induces apoptosis

**Figure 4:**
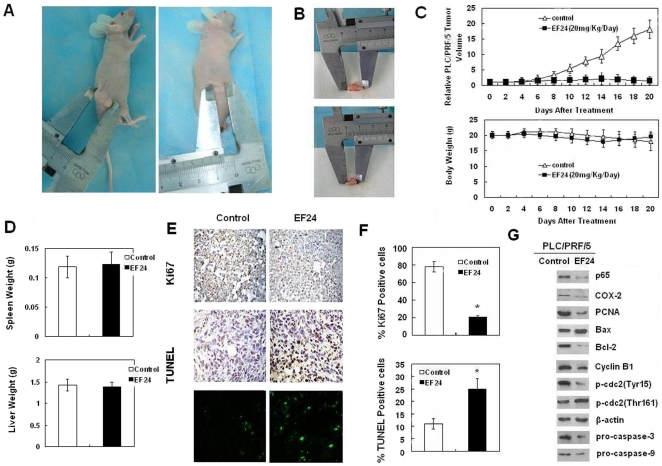
EF24 inhibits liver cancer tumor xenograft growth in vivo. (**A**) and (**B**). PLC/PRF/5 cells were injected to the flanks of nude mice and palpable tumors were allowed to develop for 7 d. Subsequently, 20 mg/kg/d of EF24 was injected daily i.p. for up to 21 d. On day 22, tumors were excised and subjected to further analyses. Tumor volumes in EF24 given mice were smaller than that of control mice. (**C**). Tumor size was measured every two days. There was a significant reduction in relative tumor volume from EF24-treated animals when compared with untreated controls. (**D**). Liver and spleen weight of the nude mice in EF24 treated and control group. (**E**) Tumor sections were stained with an anti-Ki-67 Ab to detect proliferating cells, or TUNEL agent to visualize apoptotic cells. (**F**). Cells expressing Ki-67 were counted to calculate the proliferation index, or TUNEL-positive cells. Assay was done in triplicate and p<0.05 is denoted by “*”. (**G**). Western blot analysis on the expressions of p65, Cyclin B1, p-cdc2 (Thr161), p-cdc2 (Tyr15), Bcl-2, Bax, PCNA, pro-caspase-3 and pro-caspase-9 from respective tumoral homogenate, with β-actin as protein loading control.

To evaluate the role of EF24 in tumor proliferation *in vivo*, we examined the ability of EF24 to suppress the growth of PLC/PRF/5 xenografts in nude mice. PLC/PRF/5 HCC cell derived xenograft tumors were allowed to develop and grow to a size of 100 mm^3^, following which EF24 was given i.p. for 3 weeks daily. Results suggested that EF24 could inhibit the growth of the tumor xenografts to a large extent (Fig. 4A, 4B). The time course of tumor growth (Vt/V0) is shown in Figure 4C. In general, the tumors in control group grew continuously during the experimental period whereas the tumor growth in the EF24-treated mice was suppressed significantly. However, there was no apparent change in liver weight, spleen weight, or body weight in the animals implying that EF24 is a potential therapeutic agent for treatment of liver cancers and it is relatively nontoxic to mice (Fig. 4C, D). Ki-67 staining for cell proliferation was performed in tumors removed from the animals on day 21. The relative number of ki-67 positive tumor cells was substantially less in tumors from mice treated with EF24, when compared with control tumors (Fig. 4E, F). In case of apoptosis, as shown in the representative photographs, tumor xenografts from the EF24-treated groups showed a marked increase in TUNEL-positive cells compared with the control group. Quantification of TUNEL-stained samples showed two to three fold increases (P<0.05) in the number of TUNEL-positive cells in the EF24-treated groups compared with the control group (Fig. 4E, F). The expression of p65 and NF-κB regulated gene products in liver tumor issues was also assessed by western blot, and the results revealed that EF24 decreased the expression of p65, COX-2, Cyclin B1, p-cdc2 (Tyr15), PCNA and increased the expression of p-cdc2 (Thr161) and Bax to Bcl-2 ratios (Fig. 4G).

## Discussion

HCC is a major cause of cancer death in Asia and worldwide. Most patients have inoperable disease at the time of diagnosis and need systemic therapy at some point of their disease [1], [2]. To our disappointment, no chemotherapy agent has shown reproducible benefit in controlled clinical trials and treatment outcome has remained poor due to different kinds of reasons including drug resistance and toxicity to normal cells [3], [4]. In the current study, our results presented here show that EF24 potently inhibits the proliferation of liver cancer cells, induces cell cycle arrest and apoptosis in vitro and vivo. What is more, it does not affect the proliferation of normal liver LO2 cells when treated with even a concentration at 8 µM. Besides, we report a mechanism by which EF24 potently suppresses the growth of liver cancer cells through directly down-regulating of NF-κB pathway.

Adams et al. showed that EF24 caused G2/M phase cell cycle arrest in both MDA-MB-231 human breast cancer cells and DU-145 human prostate cancer cells [28], and Selvendiran et al reported that the inhibitory effect of EF24 on cisplatin-resistant(CR) human ovarian cancer cell proliferation is associated with G2/M phase cell cycle arrest and increased G2/M checkpoint protein (pp53, p53, and p21) levels [35]. The results of our studies here have demonstrated that 2 µM EF24 could induce G2/M phase cell cycle arrest in all the five selected liver cancer cell lines. Besides, the observation of the cell cycle related protein levels suggested that, after 2 µM EF24 treatment, the cyclin B1 remained relatively unchanged. However, a decrease in the Tyr15 phosphorylation and an increase in the Thr161 phosphorylation, both of which were previously a prerequisite for the activation of cdc2 kinase at the G2/M phase, were detected along with the increase of G2/M cells. Since the cyclin B1/cdc2 kinase plays a critical role as M-phase promoting factor (MPF) in the G2/M transition, our results suggested that the unchanged cyclin B1 and the alteration in the phosphorylation status of cdc2 might render the cdc/cyclin B1 kinase active and thus, prevent the cells from completing the M phase after being treated with 2 µM EF24. However, after 4 µM EF24 treatment, the cyclin B1 protein decreased, this may suggest that different dose of EF24 may exert different effect on the G2/M cell cycle related proteins, but the cellular and molecular bases of this phenomenon remains to be clearly defined. Although most antineoplastic agents induce apoptosis in cancer cells, the mechanism by which they do so remains unclear. Previous studies have suggested that EF24 could activate caspase 3 in DU-145 and MDA-MB-231 cells[28], while Thomas *et al*. observed EF24-induced cleavage of PARP (substrate of caspase) in A549 lung cancer cells and they proposed a caspase mediated cell death/apoptosis [27]. In this study, we also showed that the activation of caspase and PARP were involved in the EF24-induced apoptosis and the general caspase inhibitor z-VAD-fmk partially blocked the EF24-induced cell death. However, even treatment with up to 50 µM z-VAD-fmk did not completely block the EF24-induced cell death, indicating that there were multiple mechanisms involving both caspase dependent and caspase-independent pathways. The data here was consistent with the results of Thomas *et al,* which suggested that EF24 could induce cell death in part through an potentially caspase independent mechanism, namely p38 activition [27].

In our study, we observed marked suppression of tumor growth in mice xenograft with EF24 treatment. There was a significant reduction in relative tumor volume in EF24-treated animals compared with untreated controls. In addition, a conspicuous suppression of proliferation was observed from the results of Ki-67 and the immunostaining for TUNEL showed that there were an increasing number of apoptosis cells in the EF24-treated animals. However, further studies are needed to confirm and extend the present study to use EF24 as an effective therapy for HCC. Absorption and pharmacokinetic properties of EF24 in particular still need to be identified in future studies, whereas the results of our preliminary studies indicate that EF24 seems to have low toxicity in liver, spleen and allows mice treated with EF24 to maintain normal weight gain [28].

Previously, Kasinski *et al.* reported a mechanism by which EF24 suppressed the NF-κB signaling pathway through direct action on the I-κB kinase (IKK) in many cancer cell types including human epithelial cervical (HeLa), breast (MDA-MB-231), human prostate (PC3), colon (HT29), human lung (A549, A460, Calu-1), non-small cell lung (H157, H358) and ovarian (1A9) cells[30]. Our results also indicate that NF-κB is constitutively active in all the human HCC cell lines examined. Besides, EF24 down-regulated the nuclear pool, or active form, of NF-κB and changed the expression of the NF-κB–regulated gene products Cyclin B1, COX-2 and Bcl-2. Moreover, EF24 could also induce apoptosis as indicated by activating caspases and PARP. The amount of required EF24 for suppressing liver cancer cell growth has been correlated with its ability to prevent the NF-κB from successfully translocating into the nucleus to exert its downstream transcription events. The EMSA results show that EF24 effectively inhibit the activition of NF-κB pathway in PLC/PRF/5, SK-HEP-1 and Hep3B cell lines with an estimated average IC50 of 2 µM. On the other hand, curcumin shows a much weaker effect on NF-κB suppression with an apparent IC50 of above 20 µM [30]. It is clear that the structural change of curcumin to EF24 drastically enhanced its anti-tumor effect as to inhibit the proliferation of liver cancer cells with an average IC50 of 2 µM. Cao *et al.* have found that the growth inhibition by curcumin in HepG2 cells was concentration and time dependent. The IC50 value for 48 h was 22.36 µg/ml (60.7 µM) [36]. Subramaniam *et al*. also provided evidence that EF24 could significantly suppress proliferation of colon cancer cell lines HCT-116 and HT-29 and a gastric cancer cell line (AGS) within a 24 h period, which continues to 72 h. More importantly, the effects were observed at a dose of 1 µM, a dose at which curcumin had no significant effect on HCT-116 cell proliferation [26]. All these results suggested that EF24 exhibited a more potent activity than curcumin both in HCC and other cancers. Besides, researches have pointed that the average IC50 values of adriamycin (ADM), 4′-epidoxorubicin (EDR), mitomycin C (MMC), cisplatin and vepesid (VP-16) for achieving the same NF-κB suppression on liver cancer cells were 0.96, 0.74, 2.81, 7.27 and 26.66 mM respectively[37]. It seems that EF24 is more potent than ADM, EDR, MMC, cisplatin and VP-16 against HCC *in vitro*. In addition, EF24 does not inhibit the proliferation of normal liver cell line LO2 and the 100 mg/kg dose did not have harmful effects. This dose was below the maximum tolerated dose (MTD) of 200 mg/kg iv (400 mg/kg ip) determined by the NCI. Besides, no liver or spleen toxicity was seen, and all of the treated mice demonstrated normal weight gain.

NF-κB activation, which is a result of underlying inflammation or the consequence of formation of an inflammatory microenvironment during malignant progression, has been observed in many solid tumors, including HCC. Most importantly, through its ability to up-regulate the expression of tumor promoting cytokines, such as IL-6 or TNF-α, and survival genes, such as Bcl-XL, NF-κB provides a critical link between inflammation and cancer [38]. HCC, which most commonly develops in the context of chronic viral hepatitis caused by either HBV or HCV infection, is considered to be a well accepted example of inflammation-linked cancer. Therefore, these evidences enlighten us to make a hypothesis that EF24 might interrupt the process of hepatocarcinogenesis through down-regulate the NF-κB pathway and further to be an agent of special therapeutic effect for hepatitis caused HCC. Besides, NF-κB plays a pivotal role in promoting chemoresistance in many solid tumors [39]. Together, these evidences suggest that EF24 might reduce the chemoresistance of HCC to some other antineoplastic agents and perhaps be used alone or in combination as a novel therapeutic regimen for HCC.

In conclusion, we might be the first to evaluate that EF24 has significant anticancer effects against human HCC. Our *in vitro* and *in vivo* studies in combination with the observation that EF24 does not affect proliferation of normal human liver cells strongly suggest that EF24 has promising potential for use as a therapeutic or chemopreventative agent for liver cancer. Similar to curcumin, EF24 also seems to have multiple molecular targets and its enhanced potency in cancer cell lines and xenograft tumors renders it is a candidate for therapeutic applications for liver cancer as well as other cancers.

## Materials and Methods

### Cell lines and reagents

EF24 was synthesized as reported by Adams et al [40]. PLC/PRF/5, Hep3B, HepG2, SK-HEP-1, Huh 7 and LO2 cell lines were purchased from the American Type Culture Collection (ATCC, Manassas, VA). Cell lines were grown as monolayers in DMEM containing 10% heat-inactivated fetal bovine serum (Gibco) and 1% antibiotic-antimycotic solution (Gibco) at 37°C in a humidified atmosphere of 5% CO_2_.

### Cell viability assays

To assess cell viability PLC/PRF/5, Hep3B, HepG2, SK-HEP-1, Huh 7 and LO2 cells were seeded onto 96-well plates at a density of 4×10^3^ per well and allowed to adhere and grow overnight in 10% heat-inactivated FBS containing DMEM. The cells were then treated with increasing doses of EF24 for 48 and 72 hours. Cell viability was measured with Cell Counting Kit-8 (CCK-8, Dojindo Molecular Technologies, Japan) according to the instruction of the manufacturer. Cell viability was expressed as a percentage of absorbance in treated wells relative to that of untreated (control) wells. Three independent experiments were done.

### Apoptosis assay

PLC/PRF/5, Hep3B, HepG2, SK-HEP-1 and Huh 7 cells treated with EF24 (2 µM, 4 µM) were harvested, washed twice with pre-chilled PBS and suspended in 1×binding buffer at a concentration of 1×10^6^ cells/mL. One hundred microliters of such solution (1×10^5^ cells) was mixed with 5 µL of Annexin V-FITC and 5 µL of Propidium Iodide (BD Biosciences, San Jose, CA, USA) according to the manufacturer's instruction. The mixed solution was gently vortexed and incubated in the dark at room temperature (25°C) for 15 min. Four hundreds microliters of 1×dilution buffer were then added to each tube and cell apoptosis analysis was performed by flow cytometry (BD FACS Calibur) within 1 h.

### Electron microscopic analysis

For electron microscopic observation, the PLC/PRF/5 cells were incubated with EF24 at the concentration (2 µM) for 48 hours. The cells cultured without treatment served as controls. We harvested floating cells together with adherent cells and centrifuged them at 2,000 rpm for 5 minutes. The cell pellets were fixed overnight at 4°C in a 0.2 M sodium cacodylate buffer containing a 2% solution of glutaraldehyde. Samples were then postfixed in cacodylate-buffered 1% osmium tetroxide, dehydrated, and embedded in Epon 812 (Nacalai Tesque, Osaka, Japan) for ultrathin sectioning. We stained the ultrathin sections with uranyl acetate and lead citrate and viewed them with an electron microscope.

### Cell Cycle Analysis

PLC/PRF/5, Hep3B, HepG2, Sk-HEP-1 and Huh 7 cells were plated at a density of 5×10^5^ per well on six-well plates. After treatment with EF24 for the indicated period, both floating and attached cells were collected into flow cytometry tubes and centrifuged at 1,000 rpm for 5 min to obtain cell pellets. The supernatant was discarded, and the cells were washed with PBS and then recentrifuged. The cells were resuspended in 100 µL PBS, 3 mL of −20°C ice-cold 70% ethanol was added, and the cells were then incubated for 1 h at 4°C. The cells were washed twice with PBS and 10 mg/mL RNase A was added. Propidium iodide was added to the tubes at a final concentration of 0.05 mg/mL and incubated at 4°C for 30 min in the dark. Cell cycle analysis was performed with a Becton Dickinson FACScan using an FL2 detector with a bandpass filter at specifications of 585 F 21 nm. In each analysis, 10,000 events were recorded. Results were analyzed with ModFit LT software (Verity Software House).

### Electrophoretic mobility shift assay (EMSA)

Nuclear extracts were obtained as for Western blotting described above. EMSAs were carried out with a 32^P^-labeled NF-κB using a Gel Shift Assay Core System kit (Promega), according to the manufacturer's instructions. After electrophoresis, gels were fixed in 10% acetic acid-30% ethanol buffer during 15 mins, and then dried under vacuum and exposed to X-ray film for three days. In some cases, a competition assay to determine sequence-specificity of protein-DNA interactions was performed by using 25-fold excess of unlabeled NF-κB -probe. For supershift analysis, anti-p65 antibodies were incubated with the nuclear extracts for 15 mins prior to the addition of the radiolabeled probe.

### Western blotting

SDS-PAGE and western blots were performed as previously described. Primary antibodies used are as follows: NF-κB (p65), Bcl-2, Bax and β-actin were purchased from Santa Cruz Biotechnology. Caspase-3, Cyclin B1, Caspase-9, PARP and cdc2 were purchased from Cell Signaling Technology. The secondary antibodies, anti-mouse IgG-HRP and anti-rabbit IgG-HRP were also purchased from Santa Cruz Biotechnology.

### Tumor Xenograft Experiments

PLC/PRF/5 hepatoma cells (2×I0^7^) were transplanted into 5-week-old athymic mice. For the treatment group, EF24 was dissolved in sodium chloride containing 1% dimethyl sulfoxide and administered by daily i.p. injection of 20 mg/kg/d for 20 days. The mice in both the treatment and control groups (n = 15 in each group) were sacrificed when snap-frozen and paraffin-embedded tumor tissue blocks had been obtained for further analysis. The body weight was recorded starting from the day of treatment, and tumor volumes were also calculated at the same time points using the following equation: tumor volume  =  length × (width)^2^ × π/6 and subsequently transformed into relative values (V) (V  =  V_t_/V_0_, where V_0_ is the tumor volume at initiation of treatment, whereas V_t_ is the tumor volume at any given day during entire treatment period). The study was approved by the Committee on the Use of Live Animals in Teaching and Research of the Harbin Medical University, Harbin, China. SYSK 2010-012.

### Ki-67 immunohistochemistry

Formalin-fixed, paraffin-embedded sections (5 µm) were stained with anti-Ki-67 (rabbit monoclonal clone SP6; NeoMarkers, Fremont, CA) antibody as described previously [12]. Results were expressed as percentage of Ki-67^+^±SE per×40 magnification. A total of 10×40 fields was examined and counted from three tumors of each of the treatment groups. The values were initially subjected to one-way ANOVA and then later compared among groups using unpaired Student's t test.

### In situ detection of apoptotic cells

Apoptotic cells were detected by terminal deoxynucleotidyl transferase–mediating dUTP nick end labeling (TUNEL) staining following the vendor's protocol. The apoptosis was evaluated by counting TUNEL-positive cells (brown-stained) as well as the total number of cells in five randomly selected fields in each sample at 400× magnification.

### Statistical analysis

All data were presented as mean ± SD of three independent experiments. Statistical significance was determined using Student's *t*-test or ANOVA. A P value of less than 0.05 was considered statistically significant.
